# Chemotherapy-induced tubulopathy: a case report series

**DOI:** 10.3389/fneph.2024.1384208

**Published:** 2024-04-11

**Authors:** Mario Alamilla-Sanchez, Juan Daniel Diaz Garcia, Valeria Yanez Salguero, Fleuvier Morales Lopez, Victor Ulloa Galvan, Francisco Velasco Garcia-Lascurain, Benjamin Yama Estrella

**Affiliations:** Department of Nephrology, November 20 National Medical Center (CMN), Mexico City, Mexico

**Keywords:** tubulopathy, electrolyte abnormalities, onconephrology, platinum, nephrotoxicity

## Abstract

Acquired tubulopathies are frequently underdiagnosed. They can be characterized by the renal loss of specific electrolytes or organic solutes, suggesting the location of dysfunction. These tubulopathies phenotypically can resemble Bartter or Gitelman syndrome). These syndromes are infrequent, they may present salt loss resembling the effect of thiazides (Gitelman) or loop diuretics (Bartter). They are characterized by potentially severe hypokalemia, associated with metabolic alkalosis, secondary hyperaldosteronism, and often hypomagnesemia. Tubular dysfunction has been described as nephrotoxic effects of platinum-based chemotherapy. We present 4 cases with biochemical signs of tubular dysfunction (Bartter-like/Gitelman-like phenotype) related to chemotherapy.

## Background

The differential diagnosis of non-proximal tubulopathies (thick ascending loop of Henle and convoluted distal tubule) is closely related to serum potassium concentrations, acid-base status, and blood pressure (1–4). The patient’s biochemical phenotype may exhibit any of the following patterns (see **Table 1**) (3, 4):

1. Hypochloremic metabolic alkalosis, hypokalemia, norm/hypotensive:
  a) Bartter syndrome.
  b) Gitelman syndrome.
  c) EAST/SeSAME syndrome.
  d) Helix syndrome.
2. Hypochloremic metabolic alkalosis, hypokalemia, arterial hypertension:
  a) Liddle’s syndrome.
  b) Apparent excess of mineralocorticoids.
3. Hyperchloremic metabolic acidosis, hyperkalemia, normo/hypotension arterial:
  a) Pseudohypoaldosteronism type 1.
4. Hyperchloremic metabolic acidosis, hyperkalemia, arterial hypertension:
  a) Pseudohypoaldosteronism type 2 (Gordon syndrome).
5. Hyperchloremic metabolic acidosis, hypo/hyperkalemia, normotension:
  a) Distal renal tubular acidosis.

**Table 1:** General characteristics of the main distal tubular syndromes.

Syndrome	Molecule and affected site	Potassium	Acid base status	Blood pressure	Others
**Bartter**	TAL: NKCC2, ROMK, ClC-Ka/b, Barttin, MAGED2	↓	Alkalosis	=/↓	↑ uCa, ↓/= sMg
**Gitelman**	DCT: NCC	↓	Alkalosis	=/↓	↓ uCa, ↓ sMg
**EAST/SeSAME**	DCT: Kir4.1	↓	Alkalosis	=/↓	↓ uCa, ↓ sMg, neurological symptoms
**Liddle**	CD: ENaC	↑	Acidosis	↑	–––
**AME**	CD: 11β-HSD2	↓	Alkalosis	↑	Growth retardation
**PHA-1**	CD: ENaC	↑	Acidosis	=/↓	CF-like pulmonary symptoms (AR) Remission after early childhood (AD)
**Gordon (PHA-2)**	CD: WNK, KLHL, Cul3	↑	Acidosis	↑	Gitelman’s “Mirror Syndrome”
**dRTA**	CD: H V-ATPase, AE1	=/↓	Acidosis	=	↓ uNH4

AME, Apparent mineralocorticoid excess; PHA, Pseudohypoaldosteronism; dRTA; Distal renal tubular acidosis; TAL, Thick ascending limb; DCT, Distal convoluted tubule; CD, Collecting duct; uCA, Urinary calcium; sMg, serum magnesium; CF, Cystic fibrosis; AR, Autosomal recessive; AD, Autosomal dominant; uNH4, urinary ammonium. = normal. ↑high. ↓low.

Therefore, the examination of urinary concentrations of potassium, chloride, magnesium, and calcium is extremely useful in the differential diagnosis of suspected tubulopathy. We present a series of 4 cases with association of tubulopathies directly related to chemotherapy.

## Case presentation

### Case 1

A 46 years-old female, with a history of gastric cancer, presented to the emergency department (ED) experiencing progressive muscle weakness and syncope. On admission, no electrocardiographic abnormalities were detected, blood pressure (BP) 122/85 mmHg, heart rate (HR) 84 bpm, Glasgow Coma Scale (GCS) score of 15 points, afebrile. She reported polyuria without dysuria, and diffuse abdominal pain. Cardiopulmonary examination revealed normal findings, with no signs of peripheral edema or hypovolemia. Two weeks before admission, she had completed her last cycle of chemotherapy with oxaliplatin.

#### Biochemical analysis

Serum creatinine 0.42 mg/dL, sodium 138 mEq/L, potassium 2.78 mEq/L, chloride 105 mmol/L, calcium 7.8 mg/dL, phosphorus 3.8 mg/dL, magnesium 1.1 mg/dL. The following urinary parameters were obtained: urinary calcium-to-creatinine ratio (uCa/uCr) 0.4 mg/mg, potassium 86.8 mEq/L, chloride 56 mEq/L, urinary potassium-to-creatinine ratio (uK/uCr) 200 mEq/g, fractional excretion of magnesium (FEMg) 6%. Venous blood gas: pH 7.53, pCO2 39 mmHg, HCO3 30.7 mmol/L, lactate 1 mmol/L, iCa 0.8 mmol/L. Ultrasound with kidneys of normal size, without nephrocalcinosis. These data reveal renal losses of potassium and magnesium, with hypercalciuria, without evidence of chloride depletion, and evidence of a mixed acid-base disorder: metabolic alkalosis with mild respiratory alkalosis.

#### Follow-up

An upper endoscopy, prompted by abdominal pain, revealed a gastric tumor that partially obstructed the lumen without active bleeding. Computed tomography evidences multiple ganglia in the retroperitoneum without evidence of urinary tract obstruction. Echocardiography was normal and the syncope was linked to electrolyte disturbance-induced arrhythmia. Oral therapy was prescribed with magnesium and potassium supplements and once the electrolyte disturbances resumed the patient was discharged from the hospital. Upon returning for a follow-up in the outpatient nephrology clinic, mild hypokalemia with normomagnesemia was found. However, due to slight dyspepsia secondary to the potassium supplements, it was decided to add 100 mg of spironolactone daily, resulting in the normalization of electrolytes with suspension of treatment after three months.

### Case 2

A 67-year-old male, diagnosed four years ago with diffuse large B-cell non-Hodgkin lymphoma, with a history of autologous hematopoietic stem cell transplantation and received the last dose of the 3rd cycle of R-DHAP (rituximab, cisplatin, dexamethasone, cytarabine). He presented to the ED due to intense muscle weakness and fatigue. Cardiopulmonary examination revealed normal findings. BP: 100/65 mmHg, HR: 98 bpm, GCS: 15 points, 36°C. Physical assessment showed slightly diminished deep patellar tendon reflexes, with no signs of neurological focalization.

#### Biochemical analysis

Serum creatinine of 0.92 mg/dL, sodium 145 mEq/L, potassium 2.9 mEq/L, calcium 8.7, phosphorus 1.8, magnesium 1.9 mg/dL. In urinary biochemistry, the following values were found: uCa/uCr 0.01 mg/mg, potassium 7.3 mEq/L, chloride 133 mEq/L, uK/uCr 40 mmol/gr, FEMg 4%. Venous blood gas: pH 7.48, pCO2 37 mmHg, HCO3 29.0 mmol/L, lactate 1.8, iCa 1.07 mmol/L. Renal ultrasound revealed normal kidneys. These data reveal renal losses of potassium and magnesium, with hypocalciuria, no chloride depletion, and mixed metabolic/respiratory alkalosis.

#### Follow-up

The exclusion of recent administration of diuretics, proton pump inhibitors, laxatives, or any dietary supplement has been confirmed. Treatment was initiated with oral supplementation with potassium and magnesium salts, leading to a gradual normalization of electrolytes. Given the unfavorable prognosis associated with the underlying pathology, the hematology department opted for a referral to palliative care. Unfortunately, the patient was lost to follow-up.

### Case 3

A 58-year-old female with squamous cell cancer affecting the middle third of the esophagus underwent endoscopic placement of a metallic prosthesis eight months ago. She received concomitant treatment involving 21 cycles of radiotherapy and four months of chemotherapy with cisplatin. The onset of symptoms occurred a few hours after the last administration of cisplatin, four days before admission. On admission to ED she exhibited generalized weakness, mild bilateral leg edema and progressively hindering her ability to walk. Normal vital signs. BP 130/75 mmHg, HR 89 rpm, GCS 15 points, afebrile.

#### Biochemical analysis

Serum creatinine 0.31 mg/dL, sodium 137 mEq/L, potassium 2.2 mEq/L, calcium 8.8 mg/dL, phosphorus 4.2 mg/dL, magnesium 0.8 mg/dL. Urinary analysis revealed: uCa/uCr 0.01 mg/mg, potassium 77 mEq/L, chloride 23 mEq/L, uK/uCr 39 mEq/gr, FEMg 26%. Venous blood gas: pH 7.53, pCO2 38 mmHg, HCO3 31.1 mmol/L, lactate 0.9 mmol/L, iCa 1.23. Renal ultrasound revealed normal kidneys. These data reveal renal losses of potassium and magnesium, with hypocalciuria, no chloride depletion, and mixed acid-base disturbance with metabolic alkalosis and respiratory alkalosis.

#### Follow-up

The initial treatment involved intravenous replacement of potassium and magnesium, followed by a transition to oral supplementation. At discharge, the regimen included potassium salts, spironolactone at 100 mg OD, and magnesium tablets, with gradual normalization of electrolytes and acid-base disturbances. Due to the poor prognosis of esophageal cancer, palliative care was incited. One month later subsequent follow-up at the outpatient nephrology clinic confirmed normal serum electrolyte level and the supplements were suspended. Unfortunately, the patient died shortly after.

### Case 4

A 38-year-old female patient with a history of left ovarian dysgerminoma began chemotherapy with BEP (Bleomycin, Etoposide, and Cisplatin), completing five cycles. However, due to cancer recurrence, a second line of therapy with a TIC regimen (Paclitaxel, Ifosfamide, and Carboplatin) was started. During her fifth cycle, routine biochemical analysis revealed various electrolytic imbalances leading to referral to the nephrology department. The patient was asymptomatic. BP 100/60, HR 79, GCS 15 points, 36°C, remaining physical examination was normal.

#### Biochemical analysis

Serum creatinine 0.63 mg/dL, sodium 142 mEq/L, potassium 3 mEq/L, calcium 9.1 mg/dL, phosphorus 3.8 mg/dL, magnesium 0.88 mg/dL. In the 24-hour urine collection: uCa/uCr 0.37 mg/mg, potassium 87 mEq/L, chloride 34 mEq/L, uK/uCr 43.3 mEq/gr, FEMg 7%. Venous blood gas: pH 7.49, pCO2 38 mmHg, HCO3 30.7 mmol/L, lactate 1.7 mmol/L, iCa 1.18 mmol/L. Renal ultrasound revealed normal kidneys. These data reveal renal losses of potassium and magnesium, with hypercalciuria, no chloride depletion, and mixed metabolic alkalosis with mild respiratory alkalosis.

#### Follow-up

The patient received oral therapy for the electrolyte disorder. Normalization of potassium and magnesium was evident a few weeks later after dose adjustments, but she continued with low-dose oral supplementation of potassium/magnesium 12 months after diagnosing tubular dysfunction, without receiving more doses of platinum-based chemotherapy.

The biochemical characteristics of the four reported cases are summarized in **Table 2**.

**Table 2:** Comparison of the 4 cases presented.

Case	Biochemical tubulopathy phenotype	Platinum-based chemotherapy	Electrolyte and acid base disturbances	Urinary ratio and fractional excretion	Tubulopathy status after platinum suspension
**1**	Bartter-like	Oxaliplatin	↓ sK, ↓ sMg, ↓ sCa Metabolic alkalosis	↑ uCa/uCr ↑ uK/uCr ↑ FEMg	Recovery
**2**	Gitelman-like	Cisplatin	↓ sK, ↓ sMg Metabolic alkalosis	↓ uCa/uCr ↑ uK/uCr ↑ FEMg	Unknown
**3**	Gitelman-like	Cisplatin	↓ sK, ↓ sMg Metabolic alkalosis	↓ uCa/uCr ↑ uK/uCr ↑ FEMg	Recovery
**4**	Bartter-like	Carboplatin	↓ sK, ↓ sMg, ↓ sCa Metabolic alkalosis	↑ uCa/uCr ↑ uK/uCr ↑ FEMg	Persistent

uCa/uCr, Urinary calcium: creatinine ratio; uK/uCr, Urinary potassium:creatinine ratio; FEMg, fractional excretion of magnesium. ↑high. ↓low.

## Discussion

Nephrotoxicity can be defined as any kidney damage caused directly or indirectly by medications. Some examples of commonly associated drugs include nonsteroidal anti-inflammatory drugs, antibiotics, and chemotherapeutic agents (5, 6). Epidemiological studies show that nephrotoxicity is the third most common cause of acute kidney injury (AKI), which has worsened in recent decades due to the more frequent use of medications with renal toxicity, studies indicate a frequency of up to 20% of all patients with AKI (5).

In the present case series report, there were several biochemical data to consider: persistent electrolyte and acid-base imbalances related to urinary loss of potassium and magnesium during subsequent evaluations, coupled with a history of platinum-based chemotherapy. This strongly indicates an acquired tubulopathy. It is crucial to confirm the absence of recent use of diuretics, proton pump inhibitors (PPI), immunosuppressants (e.g., tacrolimus, cyclosporine), antacids, laxatives, anti-EGFR/anti-VEGFR chemotherapy (e.g., cetuximab, pazopanib), or any dietary supplement such as herbal medicine, which may explain renal and/or extrarenal losses of potassium and magnesium. Tubular disorders are frequently underdiagnosed, probably due to the relative absence of specific symptoms that may be related to tubular dysfunction. Additionally, fatigue and mild muscle weakness can easily be associated with the progression of cancer or the administration of any chemotherapy. For this reason, clinician rarely requests a venous blood gas analysis or a urinary electrolyte analysis in this context. Another contributing factor to the low rate of tubular disorder diagnosis is that electrolyte abnormalities are usually managed with intravenous replacement therapy to achieve the desired electrolyte levels, without conducting a concise evaluation to determine whether the etiology is of extrarenal origin or due to a potential tubular dysfunction. This behavior can lead to high morbidity in chronically debilitated patients.

Cancer is associated with multiple fluid and electrolyte disorders. Its appearance can be related to the frequent polypharmacy: PPI, antihypertensives, diuretics, analgesics, and, of course, chemotherapy. In addition, they are frequent users of parenteral nutrition, which can cause serious potassium, phosphorous, and magnesium disorders in the context of refeeding syndrome (7) and may undergo intestinal surgeries that promote malabsorption or severe decrease in food intake, also other serious complications such as tumor lysis syndrome directly affect renal function and the body distribution of electrolytes (8).

Platinum-based drugs have as an important limitation the well-known nephrotoxicity, especially acute kidney injury and electrolyte disorders (9). The damage is mainly located in the proximal tubule (S3 portion), distal duct, and collecting duct (9). The most studied electrolyte alterations include hyponatremia (59%) and hypomagnesemia (27%) (10, 11).

Cisplatin causes dose-dependent nephrotoxicity, the tubular compartment being the most frequently affected. Within the cell, cisplatin behaves as a positively charged electrophilic molecule with high affinity for DNA, resulting in the formation of cross-links between two adjacent guanine residues, which hinders cellular synthesis and replication (12, 13). This can explain structural and functional tubular alterations leading to urinary loss of potassium, magnesium, and calcium, whose abnormalities have a prognostic effect on hospitalized patients, regardless of the cause.

Collins et al., analyzed the electronic reports of more than 911,000 patients, finding a U-shaped association between serum potassium concentrations and mortality, being cumulative for each 0.1 mEq/L change in potassium < 4 mEq/L and ≥ 5 mEq/L; the risk was higher in patients ≥ 65 years, patients with heart failure, chronic kidney disease or diabetes (14). Among patients with chronic kidney disease, where the effect of dyskalemia on mortality has been most strongly investigated, it was reported in a meta-analysis that included 11 clinical studies and 57,234 patients that a serum potassium concentration < 4 mEq/L was associated with a higher risk of all-cause mortality (HR= 1.18, 95% CI: 1.11 – 1.26), and a concentration < 3.5 mEq/L increased the risk of all-cause mortality by 105%(6). Even relatively “off-risk” serum potassium concentrations (e.g., 3.4 mEq/L) may confer an increased risk of cardiovascular complications that should be carefully considered (15).

Magnesium is another relevant cation in cellular physiology with a prognosis impact. The prevalence of hypomagnesemia is 20% in hospitalized patients, especially in patients with oncological and hematological disorders, and is related to longer hospital stay (16), although the incidence approaches 50% in patients in the ICU (17) with higher mortality compared to patients with normomagnesemia (35% vs 12%, p = 0.01) (18). Extreme values of hypomagnesemia (< 0.3 mmol/L or < 0.75 mg/dL) in hospitalized patients were evaluated by Cheminet et al., finding a prevalence of less than 0.5% (19). Of the 119 patients detected, 84% had a gastrointestinal disorder and 42% had a history of cancer. The most prescribed hypomagnesemia-related drugs were PPIs (70%), immunosuppressants (22%), platinum-based chemotherapy (17%), and diuretics (19%); the association of hypocalcemia (77%) and hypokalemia (51%) was significative (19).

Gitelman and Bartter syndromes are rare inherited tubulopathies that cause hypokalemia, metabolic alkalosis, and hypomagnesemia. Gitelman syndrome is caused by inactivating mutations in the solute transporter family 12-member 3 (SLC12A3) gen; a wide number of these mutations have been reported including nonsense, cut-and-splice site and missense mutations (20). Regarding Bartter syndrome, it is characterized by mutations in the thick ascending limb of Henle’s loop, either in NKCC2, ROMK, ClC-Ka/b, Barttin protein, CaSR, or MAGE-D2 (21) (see **Table 1**). An important characteristic in the differential diagnosis of Bartter phenotype compared to Gitelman is hypercalciuria; currently, the induction test with thiazides is no longer recommended (22). However, a genetic test is a gold standard, especially due to the overlap of biochemical phenotypes (e.g.: Gitelman and Bartter type 3). Unfortunately, genetic testing couldn’t be conducted in our institution to determine the coexistence of mutations in the co-transporters. However, the absence of electrolyte and acid-base disorders before the initiation of chemotherapy, along with the temporal association with platinum-based chemotherapy, makes the nephrotoxic effect the most plausible causal factor.

The functional tubular prognosis varies; Panichpisal et al. described the case of a patient with a 20-year follow-up who developed severe hypokalemia with metabolic alkalosis and hypocalciuria related to cisplatin administration. Although the genetic mutation status of the NCC was not determined, it is feasible that the association was causal. The same study documented 12 cases with Gitelman-like syndrome, all with persistent electrolyte abnormalities for up to 6 years (23). Tubular damage and electrolyte abnormalities may be permanent in some patients (24, 25), probably related to the toxic effect on the gene that encodes the sodium/chloride cotransporter and apoptosis of the tubular epithelial cells of the distal segment (23). To date, it is not known whether there are genetic polymorphisms associated with the risk of developing platinum-associated tubulopathy.

## Conclusion

Despite the increasing prevalence and incidence of cancer and, therefore, the prescription of new and classic chemotherapy, the diagnosis of electrolyte disorders and especially tubular dysfunction is often an underestimated complication. Its recognition will allow a multidisciplinary management that includes oncologists, nutritionists, geriatricians, and nephrologists for an accurate assessment that improves the long-term results of patients. The relevance of diagnosing these disorders can enhance integrative management, some of which have an ominous prognosis. This holds even if they are in palliative care, as it provides relief for symptoms that can deteriorate the patient’s quality of life. Additionally, this is one of the first reports to our knowledge of Bartter phenotype tubulopathy associated with platinum-based chemotherapy.

## Data availability statement

The raw data supporting the conclusions of this article will be made available by the authors, without undue reservation.

## Ethics statement

Written informed consent was obtained from the individual(s) for the publication of any potentially identifiable images or data included in this article. Written informed consent was obtained from the participant/patient(s) for the publication of this case report. 

## Author contributions

MA-S: Conceptualization, Data curation, Formal analysis, Funding acquisition, Investigation, Methodology, Project administration, Resources, Software, Supervision, Validation, Visualization, Writing – original draft, Writing – review & editing. DD: Writing – original draft, Writing – review & editing. VY: Writing – original draft, Writing – review & editing. FM: Writing – original draft, Writing – review & editing. VU: Writing – original draft, Writing – review & editing. FV: Writing – original draft, Writing – review & editing. BY: Writing – original draft, Writing – review & editing.
